# Self-Reported Walking Pace and Risk of Cardiovascular Diseases: A Two-Sample Mendelian Randomization Study

**DOI:** 10.3389/fgene.2022.871302

**Published:** 2022-06-16

**Authors:** Lu Chen, Xingang Sun, Yuxian He, Liangrong Zheng

**Affiliations:** Department of Cardiology and Atrial Fibrillation Center, The First Affiliated Hospital, School of Medicine, Zhejiang University, Hangzhou, China

**Keywords:** self-reported walking pace, atrial fibrillation, heart failure, stroke, Mendelian randomization, causal association

## Abstract

**Background:** In observational studies, the self-reported walking pace has been associated with the risk of cardiovascular diseases (CVD). However, whether those associations indicate causal links remains unclear. We performed two-sample Mendelian randomization (MR) analyses to evaluate the causal effect of walking pace on several CVD outcomes, including atrial fibrillation (AF), heart failure (HF), any stroke, ischemic stroke (IS), and IS subtypes.

**Methods:** Genetic variants associated with self-reported walking pace were selected as instrumental variables (IVs) from the latest genome-wide association studies (GWAS). Summary-level data for outcomes were obtained from the corresponding GWAS and the FinnGen consortium. The random-effects inverse variance weighted (IVW) method was used as the main MR analysis, supplemented by replication analyses using data from the FinnGen. To explore the effect of pleiotropy due to adiposity-related traits, we further conducted MR analyses by excluding the adiposity-related IVs and regression-based multivariable MR adjusting for body mass index (BMI).

**Results:** The MR results indicated significant inverse associations of self-reported walking pace with risks of AF [odds ratio (OR), 0.577; 95% confidence interval (CI), 0.442, 0.755; *p* = 5.87 × 10^−5^], HF (OR, 0.307; 95% CI, 0.229, 0.413; *p* = 5.31 × 10^−15^), any stroke (OR, 0.540; 95% CI, 0.388, 0.752; *p* = 2.63 × 10^−4^) and IS (OR, 0.604; 95% CI, 0.427, 0.853; *p* = 0.004) and suggestive inverse association of self-reported walking pace with cardioembolic stroke (CES) (OR, 0.492; 95% CI, 0.259, 0.934; *p* = 0.030). Similar results were replicated in the FinnGen consortium and persisted in the meta-analysis. However, there was no causality between walking pace and large artery stroke (OR, 0.676; 95% CI, 0.319, 1.434; *p* = 0.308) or small vessel stroke (OR, 0.603; 95% CI, 0.270, 1.349; *p* = 0.218). When excluding adiposity-related IVs and adjusting for BMI, the associations for HF and any stroke did not change substantially, whereas the associations for AF, IS, and CES were weakened.

**Conclusion:** Our findings suggested that genetically predicted increasing walking pace exerted beneficial effects on AF, HF, any stroke, IS, and CES. Adiposity might partially mediate the effect of walking pace on AF, IS, and CES.

## Introduction

Walking is an acceptable, simple, safe, and cheap kind of physical activity for the population, and it is widely promoted with its physical, mental, and social health benefits (Murtagh et al., 2015; Stamatakis et al., 2019). However, the primary focus is to increase the walking time and the number of walking steps, with the role of walking pace poorly explored (Celis-Morales et al., 2019).

As an easy and reliable measure of functional capacity, walking pace has been identified as a strong predictor of mortality and risk of cardiovascular diseases (CVD). Previous studies consistently suggested that objectively assessed walking pace was inversely associated with all-cause mortality and CVD risk (Dumurgier et al., 2009; Liu et al., 2016; Celis-Morales et al., 2019; Stamatakis et al., 2019). Recently, Timmins et al. conducted a genome-wide association study (GWAS) of self-reported walking pace and clarified the genetic associations of walking pace with a range of health outcomes (Timmins et al., 2020). They indicated that genetically predicted self-reported walking pace was associated with a lower risk of coronary artery disease (CAD) [odds ratio (OR) = 0.34, *p* = 3.1 × 10^−8^] (Timmins et al., 2020). However, whether such causal association extends to other CVD outcomes remains unknown. Notably, several observational studies showed that walking pace was significantly associated with the risk of atrial fibrillation (AF) (Mozaffarian et al., 2008; Koca et al., 2020) and heart failure (HF) (Saevereid et al., 2014) as well as stroke (Quan et al., 2020).

Mendelian randomization (MR) is a credible and powerful approach to study the causal associations between exposures and outcomes. This approach uses genetic variants which have a specific effect on a trait (exposure) as instrumental variables (IVs). Considering that genetic variants are randomly allocated at conception and thus uncorrelated with any self-adopted or environmental factors, the MR approach can minimize residual confounding (Emdin et al., 2017). Furthermore, since genetic instruments are nonmodifiable, the MR approach estimates the effect of lifelong exposure and avoids reverse causation (Sekula et al., 2016). Therefore, we conducted a two-sample MR study to investigate the causal relationships between self-reported walking pace and several CVD outcomes, including AF, HF, any stroke, ischemic stroke (IS), and IS subtypes. We also replicated the MR association of self-reported walking pace with rheumatoid arthritis (RA), which was previously reported as null-association (Timmins et al., 2020), to further test the validity of our results.

## Materials and Methods

### Study Design

This study applied a two-sample MR design to estimate the causal inference between self-reported walking pace and CVD risks. The MR study was built upon three assumptions. First, the IVs were significantly associated with exposure (self-reported walking pace). Second, the IVs should be independent of any confounders. Third, the IVs directly affected the outcome (CVD) only through exposure rather than other pathways.

### Data Sources and IV Selection

We conducted this two-sample MR study using summary-level data from previously published large-scale GWAS. The detailed descriptions of data sources are presented in Supplementary Table S1. According to the first key assumption of MR, we utilized 75 lead uncorrelated single nucleotide polymorphisms (SNPs) (linkage disequilibrium threshold of *r* ^2^ < 0.1) as the genetic instruments for self-reported walking pace, which were reported at genome-wide significance (*p* < 5 × 10^−8^) in the GWAS utilizing the United Kingdom Biobank data (Timmins et al., 2020). This study included 450,967 participants (average age 58 and 45.7% of them were male) who reported the walking pace as a category phenotype: “slow,” “steady/average,” or “brisk,” and this phenotype was coded 0, 1, and 2, respectively, to further use a linear mixed model with covariates for age, sex, genotyping array, and 20 principal components of ancestry implemented (Timmins et al., 2020). F-statistic for each IV was calculated to evaluate the strength of the relationship between IV and self-reported walking pace (Supplementary Table S1). SNP with F-statistic >10 was generally recommended as an indication of strong IV (Burgess and Thompson, 2011). CVD outcomes included AF, HF, any stroke, IS, and IS subtypes in this study. The summary-level data for these outcomes were obtained from GWAS meta-analysis (Nielsen et al., 2018), the HERMES consortium (Shah et al., 2020), the MEGASTROKE consortium (Malik et al., 2018), and the FinnGen consortium (FinnGen Consortium, 2021). If IVs were not available in the outcome database, proxy SNPs would be searched online (http://snipa.helmholtz-muenchen.de/snipa3/) to replace them, and the proxy searching results are presented in Supplementary Table S2. Moreover, for each CVD outcome, IVs directly associated (*p* < 5 × 10^−8^) with each CVD outcome were excluded from our analyses. After extracting the summary-level data, we further harmonized the exposure and outcome data to ensure the effect of an SNP on the exposure and the effect of that SNP on the outcome each corresponding to the same allele.

### Statistical Analysis

The multiplicative random-effects inverse variance weighted (IVW) method was used as the main MR method to assess the causal inferences between self-reported walking pace and CVD outcomes. The replication analyses were also performed to validate the reliability of the results using summary-level data from the FinnGen consortium (Table 1). Then, the fixed-effects meta-analysis method was used to combine the MR estimates from different data sources. Several complementary MR analyses were used to provide credible estimates and detect pleiotropy, including the weighted median (Bowden et al., 2016), MR-robust-adjusted profile score (MR-RAPS) (Zhao et al., 2018), MR-Egger (Bowden et al., 2015), and the MR-pleiotropy residual sum and outlier (MR-PRESSO) (Verbanck et al., 2018) methods. The weighted median method assumed that ≥50% of the weight was derived from valid SNPs (Bowden et al., 2016). The MR-RAPS corrected for horizontal pleiotropy in the IVW analysis (Zhao et al., 2018). The MR-Egger generated MR estimates after correction for pleiotropy (Bowden et al., 2015). Moreover, the MR-PRESSO provided robust MR estimates by excluding the identified outliers (Verbanck et al., 2018). Besides, we chose RA as a negative control outcome which was reported by a previous MR study that genetically predicted that self-reported walking pace was not causally associated with RA (OR = 0.59, *p* = 0.172) (Timmins et al., 2020). Information on SNP–rheumatoid arthritis associations was obtained from large-scale GWAS conducted by Okada et al. (2014) (Table 1). We further undertook multiple sensitivity analyses to examine the robustness of the results and avoid possible pleiotropy bias. First, we calculated the I^2^ index and displayed the scatter plots and funnel plot to identify the possible heterogeneity among IVs. An I^2^ index >25% was deemed as the marker of heterogeneity (low: 25% < I^2^ < 50%; moderate: 50% < I^2^ < 75%; high: I^2^ > 75%). Second, the intercept test from the MR-Egger method was conducted to measure pleiotropy (Bowden et al., 2015). Third, we performed the leave-one-out analysis to evaluate the consistency of causal associations and whether any individual SNP affected the results.

**TABLE 1:** Characteristics of the data sources.

Trait	Data source	Sample size (case/control)	Ancestry	Use	Phenotype/case definition	Covariates adjusted in GWAS
Self-reported walking pace	Timmins et al. (2020)	450,967	European	Exposure	Category phenotype: “slow,” “steady/average,” or “brisk"	Age, sex, genotyping array, and the first 20 principal components of ancestry
Atrial fibrillation	Nielsen et al. (2018)	60,620/970,216	European	Outcome	ICD-10 code I48 or ICD-9 code 427.3 or 12-lead ECG at the examinations	Birth year, sex, genotype batch, and principal components 1–4
Atrial fibrillation	FinnGen Consortium (2021)	22,068/116,926	European	Outcome for replication analyses	ICD-10 code I48	Sex, age, 10 principal components, and genotyping batch
Heart failure	HERMES Shah et al. (2020)	47,309/930,014	European	Outcome	Self-reported, physician diagnosis, or the ICD-9 or ICD-10 codes for discharge diagnosis	Age, sex, and principal components in individual studies, where applicable
Heart failure	FinnGen Consortium (2021)	23,397/194,811	European	Outcome for replication analyses	ICD-10 code I11.0, I13.0, I13.2, I50	Sex, age, 10 principal components, and genotyping batch
Any stroke	MEGASTROKE Malik et al. (2018)	40,585/406,111	European	Outcome	Rapidly developing signs of focal (or global) disturbance of cerebral function, lasting more than 24 h or leading to death with no apparent cause other than that of vascular origin	Age and sex
Any stroke	FinnGen Consortium (2021)	18,661/162,201	European	Outcome for replication analyses	ICD-10 code I60, I61, I62, I63, I64, G45	Sex, age, 10 principal components, and genotyping batch
Ischemic stroke	MEGASTROKE Malik et al. (2018)	34,217/406,111	European	Outcome	IS was defined based on clinical and imaging criteria	Age and sex
Ischemic stroke	FinnGen Consortium (2021)	10,551/202,223	European	Outcome for replication analyses	ICD-10 code I63, I64	Sex, age, 10 principal components, and genotyping batch
Large artery stroke	MEGASTROKE Malik et al. (2018)	4,373/146,392	European	Outcome	Trial of Org 10172 in Acute Stroke Treatment (TOAST) criteria	Age and sex
Small vessel stroke	MEGASTROKE Malik et al. (2018)	5,386/192,662	European	Outcome	Trial of Org 10172 in Acute Stroke Treatment (TOAST) criteria	Age and sex
Cardioembolic stroke	MEGASTROKE Malik et al. (2018)	7,193/204,570	European	Outcome	Trial of Org 10172 in Acute Stroke Treatment (TOAST) criteria	Age and sex
Rheumatoid arthritis	Okada et al. (2014)	14,361/42,923	European	Negative control outcome	1987 criteria of the American College of Rheumatology for RA diagnosis or were diagnosed as RA by a professional rheumatologist	Top 5 or 10 principal components
Body mass index	Hoffmann et al. (2018)	334,487	Mixed (94.3% European)	Confounder for MVMR analyses	Weight (kg)/height (m)^2^	Age, sex, and ancestry

Abbreviations: GWAS, genome-wide association studies; ICD, International Classification of Diseases; ECG, electrocardiogram; MVMR, regression-based multivariable MR.

Since the selected IVs should be independent of any confounders and exert an effect on the outcome only through the exposure, we further conducted analyses to explore the effect of pleiotropy due to adiposity-related traits, as several of the selected IVs are associated with the body mass index (BMI) and weight according to the PhenoScanner (http://www.phenoscanner.medschl.cam.ac.uk) and Open Target Genetics platform (https://genetics.opentargets.org) (Supplementary Tables S3, S4). First, MR analyses were re-conducted after excluding these SNPs which were previously associated with BMI or weight. Second, we additionally implemented the regression-based multivariable MR (MVMR) according to Yavorska and Burgess (2017) to obtain the direct MR estimates independent of BMI. The summary statistic data for genetic associations of IVs with BMI were extracted from large meta-analysis of GWAS (Hoffmann et al., 2018).

We used a web-based application (https://cnsgenomics.com/shiny/mRnd/) to calculate the *post hoc* statistical power. Supplementary Table S5 shows the required minimum effect of self-reported walking pace on CVD outcomes to reach 80% statistical power based on the variance explained by the selected IVs (∼0.92%) and sample sizes of outcomes.

The results were presented as ORs and corresponding 95% confidence intervals (CIs), which were scaled per category increase in the self-reported walking pace (slow, steady/average, and brisk). The Bonferroni method was used to account for multiple comparisons (eight outcomes). The associations with *p*-values < 0.006 (0.05/8) were regarded as statistically significant associations, and associations with *p*-values between 0.05 and 0.006 were considered as suggestive associations. The summary-level data used in this study were publicly available, and no ethics approval or informed consent was required. All analyses were conducted in R foundation (Version 4.0.2) by using package TwosampleMR (Hemani et al., 2018), MR-PRESSO (parameters: NbDistribution = 2,500, SignifThreshold = 0.05) (Verbanck et al., 2018), and Mendelian randomization (Yavorska and Burgess, 2017).

## Results

**FIGURE 1:**
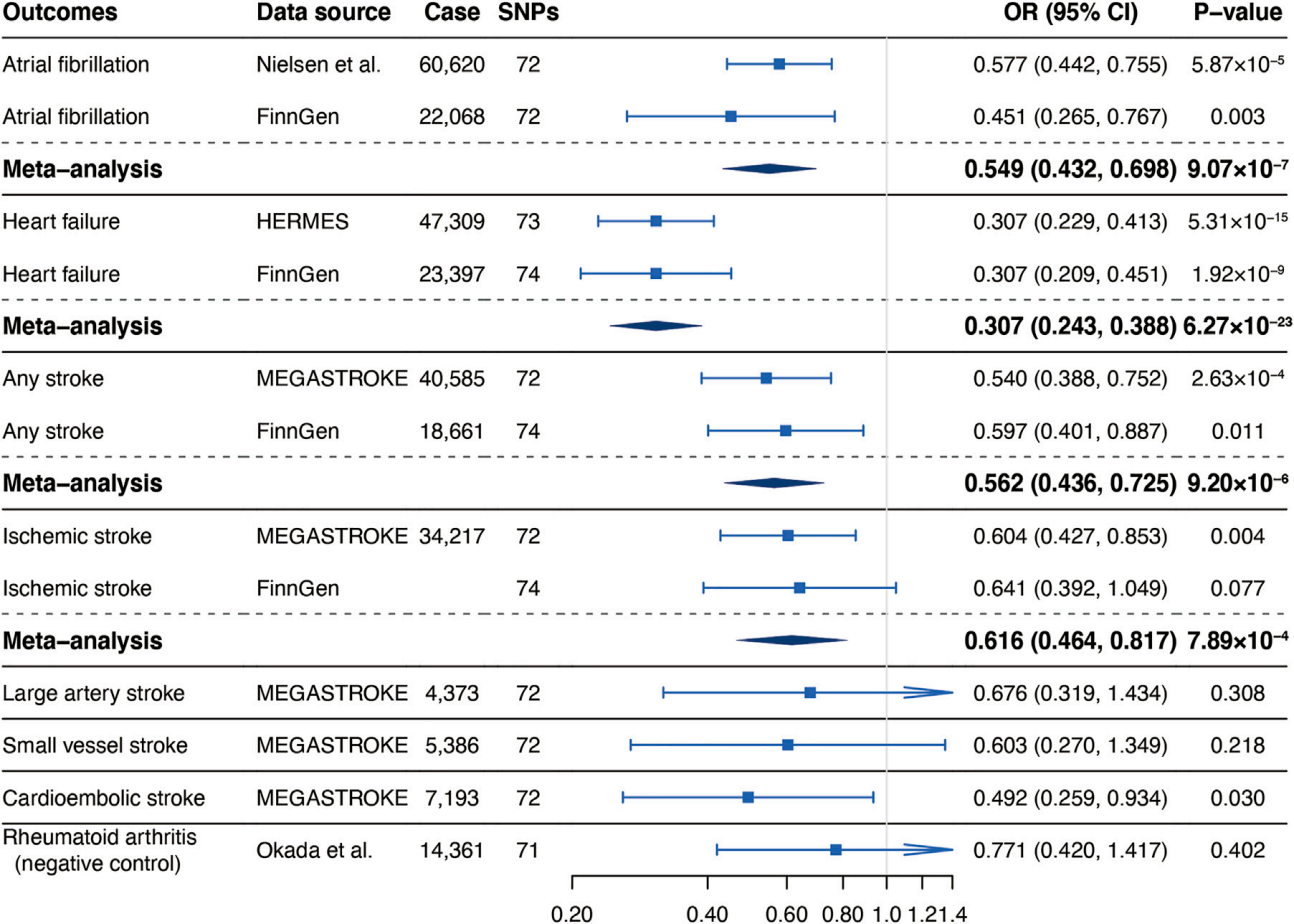
Associations of genetically predicted self-reported walking pace with risk of cardiovascular diseases. Abbreviations: SNPs, single nucleotide polymorphisms; OR, odds ratio; CI, confidence interval; HERMES, Heart Failure Molecular Epidemiology for Therapeutic Targets.

Our MR analyses found significant inverse associations of self-reported walking pace with risks of AF in the GWAS meta-analysis of Nielsen et al. (OR, 0.577; 95% CI, 0.442, 0.755; *p* = 5.87 × 10^−5^), HF in the HERMES (OR, 0.307; 95% CI, 0.229, 0.413; *p* = 5.31 × 10^−15^), any stroke (OR, 0.540; 95% CI, 0.388, 0.752; *p* = 2.63 × 10^−4^) and IS (OR, 0.604; 95% CI, 0.427, 0.853; *p* = 0.004) and suggestive inverse association of self-reported walking pace with cardioembolic stroke (CES) (OR, 0.492; 95% CI, 0.259, 0.934; *p* = 0.030) in the MEGASTROKE (Figure 1). There was no causal association between genetically predicted walking pace and large artery stroke (LAS) (OR, 0.676; 95% CI, 0.319, 1.434; *p* = 0.308) or small vessel stroke (SVS) (OR, 0.603; 95% CI, 0.270, 1.349; *p* = 0.218) (Figure 1). The significant associations for AF and HF were replicated in the FinnGen consortium and persisted in the meta-analysis (Figure 1). Although the association for any stroke was attenuated and the association for IS was not replicated in the FinnGen dataset, the meta-analysis supported the causal associations (Figure 1).

Sensitivity analyses showed that there was low heterogeneity for AF, HF, any stroke, and SVS indicated by the I^2^ index, scatter plot, or funnel plot (I^2^ = 42.3% for AF, I^2^ = 37.2% for HF, I^2^ = 37.2% for any stroke, and I^2^ = 37.2% for SVS, respectively, Supplementary Table S6; Supplementary Figures S1–S4). No heterogeneity was observed for IS, LAS, or CES (I^2^ = 24.8% for IS, I^2^ = 2.8% for LAS, and I^2^ = 19.4% for CES, respectively, Supplementary Table S6; Supplementary Figures S5–S7). The MR-Egger intercept test showed no evidence of horizontal pleiotropy in the analyses (Supplementary Table S6). Additionally, the leave-one-out analysis demonstrated that the overall causal effects for most CVD outcomes were not driven by any individual SNP (Supplementary Figures S1–S7), except that the potential causal relationship between walking pace and CES was modestly influenced by rs273512 (Supplementary Figures S7C).

We further conducted other MR methods to evaluate the robustness of our results. The MR estimates remained stable and robust in the weighted median and MR-RAPS methods. Nevertheless, the estimates deviated toward a null association in MR-Egger analyses except for any stroke (Table 2). Two SNPs (rs12127073 and rs9972653) for AF and two SNPs (rs7924036 and rs9972653) for HF were identified as outliers by the MR-PRESSO method (Supplementary Figures S1, S2). However, excluding those outliers did not substantially change the results (Table 2).

**TABLE 2:** Sensitivity analyses of the associations of genetically predicted self-reported walking pace with cardiovascular diseases.

Outcome	MR method	SNP	OR (95% CI)	*p*-value
AF (Nielsen et al.)	IVW	72	0.577 (0.442, 0.755)	5.87 × 10^−5^
	Weighted median	72	0.643 (0.476, 0.868)	3.94 × 10^−3^
	MR-RAPS	72	0.564 (0.434, 0.733)	1.81 × 10^−5^
	MR-Egger	72	1.041 (0.347, 3.117)	0.944
	MR-PRESSO	70	0.569 (0.449, 0.720)	1.34 × 10^−5^
HF (HERMES)	IVW	73	0.307 (0.229, 0.413)	5.31 × 10^−15^
	Weighted median	73	0.299 (0.209, 0.427)	3.38 × 10^−11^
	MR-RAPS	73	0.308 (0.232, 0.409)	4.59 × 10^−16^
	MR-Egger	73	0.479 (0.131, 1.748)	0.269
	MR-PRESSO	71	0.311 (0.237, 0.408)	2.97 × 10^−12^
AS (MEGASTROKE)	IVW	72	0.540 (0.388, 0.752)	2.63 × 10^−4^
	Weighted median	72	0.567 (0.370, 0.869)	0.009
	MR-RAPS	72	0.548 (0.373, 0.805)	0.002
	MR-Egger	72	0.216 (0.050, 0.939)	0.045
	MR-PRESSO	NA	NA	NA
IS (MEGASTROKE)	IVW	72	0.604 (0.427, 0.853)	0.004
	Weighted median	72	0.560 (0.355, 0.883)	0.013
	MR-RAPS	72	0.610 (0.422, 0.881)	0.009
	MR-Egger	72	0.324 (0.069, 1.516)	0.157
	MR-PRESSO	NA	NA	NA
LAS (MEGASTROKE)	IVW	72	0.676 (0.319, 1.434)	0.308
	Weighted median	72	0.496 (0.172, 1.434)	0.196
	MR-RAPS	72	0.713 (0.315, 1.614)	0.417
	MR-Egger	72	0.181 (0.006, 5.380)	0.327
	MR-PRESSO	NA	NA	NA
SVS (MEGASTROKE)	IVW	72	0.603 (0.270, 1.349)	0.218
	Weighted median	72	0.647 (0.221, 1.897)	0.428
	MR-RAPS	72	0.690 (0.289, 1.645)	0.402
	MR-Egger	72	0.165 (0.005, 5.891)	0.327
	MR-PRESSO	NA	NA	NA
CES (MEGASTROKE)	IVW	72	0.492 (0.259, 0.934)	0.030
	Weighted median	72	0.430 (0.189, 0.979)	0.044
	MR-RAPS	72	0.490 (0.247, 0.973)	0.042
	MR-Egger	72	0.074 (0.004, 1.341)	0.082
	MR-PRESSO	NA	NA	NA
RA (Negative control)	IVW	71	0.771 (0.42, 1.417)	0.402
	Weighted median	71	1.000 (0.494, 2.023)	1.000
	MR-RAPS	71	0.771 (0.401, 1.481)	0.434
	MR-Egger	71	0.747 (0.038, 14.611)	0.848
	MR-PRESSO	NA	NA	NA

Abbreviations: MR, Mendelian randomization; SNP, single nucleotide polymorphism; OR, odds ratio; CI, confidence interval; AF, atrial fibrillation; IVW, inverse variance weighted; MR-RAPS, MR-robust-adjusted profile score; MR-PRESSO, MR-pleiotropy residual sum and outlier; HF, heart failure; AS, any stroke; IS, ischemic stroke; LAS, large artery stroke; SVS, small vessel stroke; CES, cardioembolic stroke; RA, rheumatoid arthritis; NA, not available.

**FIGURE 2:**
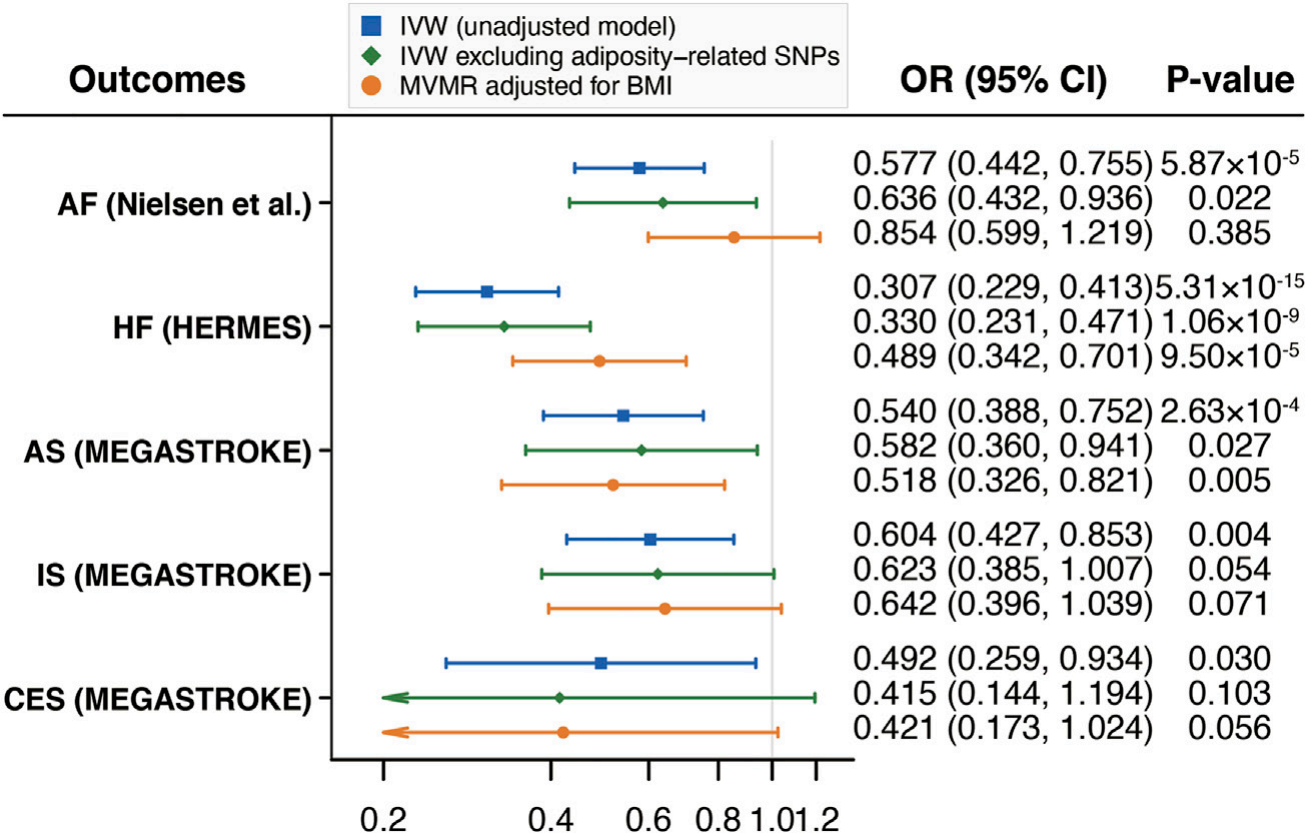
Mendelian randomization analyses of the associations of self-reported walking pace with CVD excluding adiposity-related SNPs and adjusting for BMI. Abbreviations: CVD, cardiovascular diseases; SNPs, single nucleotide polymorphisms; BMI, body mass index; IVW, inverse variance weighted; MVMR, multivariable Mendelian randomization; OR, odds ratio; CI, confidence interval; HERMES, Heart Failure Molecular Epidemiology for Therapeutic Targets; AF, atrial fibrillation; HF, heart failure; AS, any stroke; IS, ischemic stroke; CES, cardioembolic stroke.

After excluding adiposity-related SNPs and adjusting for BMI in the MVMR analyses, the associations did not change substantially for HF and any stroke (Figure 2). There was suggestive evidence of an association between walking pace and AF after excluding adiposity-related SNPs but the association was weakened after adjusting for BMI. We also found that the estimates on IS and CES were fully attenuated after excluding adiposity-related SNPs or adjusting for BMI although the trend of inverse associations remained (Figure 2).

## Discussion

We conducted a two-sample MR study to investigate whether genetically determined walking pace was causally associated with the CVD risks. Our findings indicated that genetically predicted increasing walking pace was related to lower risks of AF, HF, any stroke, IS, and CES.

A previous study showed that walking pace was significantly associated with the risk of AF, with individuals with the pace of 2–3 miles per hour (mph) and >3 mph associated with 32% (multivariable relative risk, 0.68; 95% CI, 0.59, 0.77; *p* < 0.001) and 41% (multivariable relative risk, 0.59; 95% CI, 0.48,0.74; *p* < 0.001) lower risk of AF, respectively, compared to pace <2 mph (Mozaffarian et al., 2008). However, using the Multi-Ethnic Study of Atherosclerosis Typical Week Physical Activity Survey, Mokhayeri et al. (2017) concluded that walking pace was not associated with AF incidence. These inconsistent results from observational studies might be biased by insufficient follow-up duration and small sample size as well as unmeasured confounding factors, such as age, gender, and study populations. In this study, we performed MR analyses with summary-level data derived from large-scale GWAS meta-analysis conducted by Nielsen et al. and found that brisk walking pace was associated with a reduced risk of AF (OR, 0.577; 95% CI, 0.442, 0.755; *p* = 5.87 × 10^−5^), which was absent of confounding bias. Additionally, the significant association was replicated both in the FinnGen consortium and the meta-analysis combining these two datasets, indicating the reliability of our results and suggesting that walking faster could be a protective factor for AF.

Studies have demonstrated that physical activity was related to a reduced risk of developing HF (Djoussé et al., 2009). However, little is known about the effect of walking pace on the risk of HF. Pulignano et al. (2016) showed that walking pace was independently associated with hospitalization for HF [hazard ratio (HR), 0.697; 95% CI, 0.547, 0.899; *p* = 0.004], and walking pace could improve risk stratification in older HF patients evaluated using the Cardiac and Comorbid Conditions Heart Failure score. Another prospective study suggested an inverse dose-response relationship between walking pace and the risk of HF, with moderate and high walking pace correlated with adjusted HRs of 0.53 (95% CI, 0.43, 0.66) and 0.30 (95% CI, 0.21, 0.44), respectively (Saevereid et al., 2014). Consistent with these two studies, we found that brisk walking pace was inversely associated with HF risk both in the primary analysis (OR, 0.307; 95% CI, 0.229, 0.413; *p* = 5.31 × 10^−15^) and the replication analysis (OR, 0.307; 95% CI, 0.209, 0.451; *p* = 1.92 × 10^−9^).

Regarding the relationship between walking pace and stroke, the findings of epidemiologic studies were inconclusive, and the data specific to stroke subtypes were limited. Several prospective studies suggested that a slow walking pace was associated with a higher risk of stroke (McGinn et al., 2008; Hayes et al., 2020), with HRs ranging from 1.45 to 1.69. A recent meta-analysis reported that individuals in the fastest walking pace category had a 44% lower risk of stroke than those in the slowest walking pace category [risk ratio (RR), 0.56; 95% CI, 0.48, 0.65] (Quan et al., 2020). They also observed a linear dose-response relationship, with the risk of stroke decreased by 13% for every 1 km/h increment in walking pace. The present MR study confirmed and extended the results of those studies, suggesting an inverse causal association between self-reported walking pace and the risk of any stroke, IS, and CES, with ORs being 0.540 for any stroke, 0.604 for IS, and 0.492 for CES. By contrast, such correlation was not observed in two other observational studies conducted by Jefferis et al. (2014) and Camargo et al. (2016). It is noteworthy that Jefferis et al. only examined the effect of walking pace on the risk of stroke in men, which limited the generalizability of the results. In addition, confounders might occur, which could influence the outcome variables. Thus, more studies are warranted to confirm our results, especially those specific to stroke subtypes.

The causal estimates of walking pace on AF, IS, and CES were attenuated after excluding adiposity-related SNPs or adjusting for BMI, which suggested that the observed associations might be partially mediated through adiposity, a well-known risk factor for CVD (Khan et al., 2018). As adiposity management is an effective way to reduce CVD risk, increasing walking pace thus may be a potential way to reduce CVD risk. Whether walking pace could affect adiposity is uncertain, only a weak correlation of walking pace with BMI was observed by Halliday et al. (2020). Further studies are warranted to elucidate the association between walking pace and adiposity. In addition, it is notable that the causal associations for HF and any stroke remained after excluding adiposity-related SNPs and adjusting for BMI, indicating that increasing walking pace has a direct protective effect on HF and stroke.

This study has important implications for physical activity recommendations, as CVD remains the leading cause of mortality worldwide (Townsend et al., 2016). Our results showed that brisk walking pace was causally correlated with the reduced risk of several outcomes of CVD, including AF, HF, any stroke, IS, and CES, suggesting that guidelines should encourage people to increase their walking pace and maximize its health benefits in preventing CVD. In addition, walking is a safe, cost-effective, and accessible physical activity. A pace change may be more feasible than increasing the walking duration for those with adequate physical capacity (Stamatakis et al., 2019).

The present study had several strengths. We used the MR method to elucidate the causal associations between self-reported walking pace and several CVD outcomes, which avoided residual confounding bias and reverse causation. We also applied comprehensive sensitivity analyses and MVMR to assess the robustness of our study and minimize the potential pleiotropic effect. Moreover, we implemented summary-level statistics for outcomes from two different data sources. The results of the replication analyses were mainly consistent with our main results, supporting that our findings were reliable.

Nonetheless, some limitations should be considered. We only used self-reported data on walking pace using the ACE touchscreen questionnaire due to a lack of objectively measured summary-level data, which might lead to misclassification bias. However, it was reported that self-reported walking pace was highly correlated with actual and measured walking pace (Zeki Al Hazzouri et al., 2017). In addition, self-reported walking pace is considered a general indicator of an individual’s perceived health and physical frailty in the elderly (Binotto et al., 2018; Timmins et al., 2020). However, we did not perform additional analyses to check whether other conditions exerted an effect on CVD through walking pace. Further studies can take self-reported walking pace into account when they infer causal associations between potential risk factors and CVD. In addition, most of the individuals involved in our study were of European ancestry, limiting our findings’ generalizability to other ancestries. Pleiotropy is a potential limitation in any MR analyses. However, we detected no evidence of pleiotropy using the MR-Egger regression method. To minimize potential pleiotropy bias, we further re-conducted MR analyses by excluding adiposity-associated SNPs and adjusting for BMI. Last, selection bias has been increasingly recognized as a limitation of MR, mainly due to limited representativeness of the selected sample, attrition from an initially representative sample, and selecting sample strongly surviving the exposure (Schooling et al., 2020; Mendelian randomization, 2022). Since previous studies have reported that walking pace strongly affects survival (Celis-Morales et al., 2019; Timmins et al., 2020) and GWAS participants are typically recruited in middle age or older, selection bias can be present in our study.

## Conclusion

In conclusion, this two-sample MR study showed that genetically predicted increasing walking pace was related to lower risks of AF, HF, any stroke, IS, and CES, suggesting that physical activity recommendations should encourage people to increase their walking pace its in preventing CVD.

## Data Availability

The original contributions presented in the study are included in the article/Supplementary Material. Further inquiries can be directed to the corresponding author.
